# Testing Demands and Resources as Determinants of Vitality among Different Employment Contract Groups. A Study in 30 European Countries

**DOI:** 10.3390/ijerph16244951

**Published:** 2019-12-06

**Authors:** Jari J. Hakanen, Annina Ropponen, Hans De Witte, Wilmar B. Schaufeli

**Affiliations:** 1 Workability and Work Careers, Finnish Institute of Occupational Health, BOX 40, 00032 Helsinki, Finland; annina.ropponen@ttl.fi; 2 Research Unit Occupational & Organizational Psychology and Professional Learning, KU Leuven, 3000 Leuven, Belgium; hans.dewitte@kuleuven.be (H.D.W.); w.schaufeli@uu.nl (W.B.S.); 3 Optentia Research Focus Area, North-West University, Vanderbijlpark 1900, South Africa; 4 Department of Social, Health & Organizational Psychology, Utrecht University, 3584 CH Utrecht, The Netherlands

**Keywords:** employment contracts, vitality at work, well-being, work engagement, exhaustion, burnout, job demands–resources model, Europe

## Abstract

The aim of this study was to investigate the relative importance of four job demands and five job resources for employee vitality, i.e., work engagement and exhaustion, in three different employment groups: permanent, temporary and temporary agency workers. We employed data from the sixth European Working Conditions Survey (EWCS) collected in 2015 comprising 28,042 employees from 30 European countries. We used linear regression analyses and dominance analysis (DA). The results showed minor mean differences in work engagement and exhaustion and that temporary agency workers had the highest job insecurity and lowest job control. The associations between job resources and job demands, and work engagement and exhaustion of the groups, did not differ considerably. DA showed that in all three employment groups, job feedback made the strongest contribution to work engagement and workload to exhaustion. In addition, among the temporary agency workers, supervisor support contributed to work engagement and job control (negatively) to exhaustion more than in the other groups. This study suggests that the key determinants of vitality at work may be similar, regardless of contract, and that to have sustainably performing vital workers, organizations should focus on enabling job feedback and preventing high workload in all employment groups.

## 1. Introduction

Recent decades have witnessed the growth of different types of non-traditional employment [1]. For instance, in most European countries, in addition to open-ended permanent contracts, temporary (fixed-term) contracts and temporary agency employment contracts became common in the late 1970s and the late 1990s, respectively [2]. Possible reasons for this development are global competition, technological innovations, and the need to reduce labor cost, which in many organizations has meant an increased use of temporary workforce as a strategy for increasing flexibility and reducing costs and administrative complexity in order to move toward leaner, flatter organizations [3,4]. Organizations also hire temporary employees because of the need to balance peaks in demand and to replace permanent workers [5]. Over time, non-traditional employment has become more common and taken on new forms, and this poses challenges for organizations to keep their employees sustainably healthy, engaged and productive, regardless of the contract type they have. 

The differences between the quality of working conditions and the levels of well-being in different employment contract groups have been widely studied, but the results remain inconclusive [1,6,7,8,9]. Less attention has been paid to the comparison of different employment contract groups from the perspective of whether different work characteristics are equally important for vitality at work for employees in different employment groups. By vitality at work, we refer to mental (in contrast to physical) energy: high levels of vitality are characterized by work engagement and low levels of vitality by exhaustion. In this study, we aimed to compare the antecedents of vitality at work among permanent, temporary and temporal agency workers. To do this, we used representative data from 30 countries from the sixth European Working Conditions Survey (EWCS), carried out in 2015. In addition, we utilized the job demands–resources (JD-R) model [10,11] to conceptualize and investigate two opposite poles of vitality at work—work engagement and exhaustion—and their determinants (job demands and job resources). Through this study, we hope to be able to identify pathways towards sustainable performance and to contribute to the psychological research on different types of employment contracts. Previous studies comparing different contract groups have often suffered from a lack of theoretical framework [4] and have included employees from only one country (and often from one or only a few organizations), although many country-level factors such as legislation may have confounded the study findings [4].

### 1.1. Working Conditions and Well-Being among Temporary Agency, Temporary, and Permanent Workers

In recent decades, employment contracts have become diversified and non-traditional employment of limited duration have become more common. The percentage of temporary employees relative to the total workforce has been estimated to be 12% in OECD countries and 14% in EU countries, ranging from 1% in Romania to 27% in Spain [12]. Temporary workers are typically hired directly by the employing organization. A more recent type of non-traditional contract is temporary agency employment, wherein an employee is hired by an agency to work for a client [1]. This is often called a ‘triangular’ employment relationship, as the contract involves the worker, the staffing agency and the client organization [13]. The number of temporary agency workers accounts for approximately 2% of all employment in the world, also in Europe and the USA [14].

Because temporary workers are often hired to work in the periphery of the organization and are considered replaceable (precarious work), they are often seen as having fewer job resources such as autonomy, job control, ability to use their skills, and opportunities to learn, grow and participate in decision making [5]. Indeed, several studies support these assumptions: temporary jobs have been associated with poorer job content in terms of, for example, less autonomy, control and skill variety and training, and lower pay [4,5,6]. In addition to the lack of job resources, temporary workers have also been found to be subject to more job demands than permanent workers, such as job insecurity and physical demands and burdens in the form of noise and difficult postures [1]. These are all factors that are likely to compromise vitality experienced at work.

As temporary and temporary agency workers often suffer from poorer working conditions, particularly from lacking job resources, one would assume that they would also suffer from poorer vitality, well-being, health and work performance. However, here, the evidence is even more inconclusive and contradictory [1,5,7]. Several studies have reported lower levels of well-being and health among temporary than permanent workers: for example, higher stress [15], lower job satisfaction [3,4,16] and poorer psychological well-being [16]. On the other hand, many studies have also indicated that temporary employment may not be so harmful to employees and may in fact be related to, for example, fewer health problems and sickness absences [9,17]. These inconsistent findings concern not only working conditions, well-being and health, they also concern other aspects of sustainable performance such as job performance and productive behaviors [4]. 

Most previous studies comparing different employment contract groups have focused on investigating how contract type is related to the quality of working conditions, well-being and health. However, less is known about whether working conditions associate similarly or differently with the outcomes in different contract groups. In the present study of employees from 30 European countries, we aimed to investigate how various job demands and job resources relate to vitality at work in different employment groups, and whether the same or different job demands and resources are more important for vitality in these groups. 

### 1.2. Vitality at Work from the Perspective of the Job Demands–Resources (JD-R) Model

Vitality has been characterized as a state of positive arousal and energy, and a sense of being alive, passionate, and excited [18,19]. It is also a lack of fatigue [19,20]. 

We examine vitality at work using the job demands–resources (JD-R) model [10,11,21] to investigate work engagement and (lack of) exhaustion—two opposite indicators of vitality at work—and their antecedents (job demands and job resources) among permanent, temporary and temporary agency employees. We consider the JD-R model useful in this context as it simultaneously takes into account the determinants of vitality at work and suggests pathways towards sustainable performance: A positive pathway via job resources and work engagement boosting sustainable performance and a negative pathway that may undermine sustainable performance via job demands and exhaustion/burnout. 

The JD-R model splits work characteristics into two broad categories: job demands and job resources [10,11,21]. Job demands refer to the aspects of a job that require sustained physical and/or psychological effort and are therefore associated with certain physiological and/or psychological costs that may lead to exhaustion and finally to burnout. Job resources, in turn, refer to the physical, psychological, social, or organizational aspects of a job that are important for achieving work goals; stimulate personal growth, learning and development; and may also reduce job demands. Hence, job resources have both extrinsic and intrinsic motivational potential that may not only enhance work engagement but also negatively impact exhaustion (burnout). 

In the past 20 years, work engagement has been the most widely studied construct illustrating vitality and positive energy at work. Work engagement has been defined as a positive fulfilling state at work that consists of feeling vigorous, dedicated, and absorbed [22]. Vigor refers to high levels of energy and mental resilience while working, the willingness to invest effort in one’s work, and persistence in the face of difficulties. Dedication refers to a sense of significance, enthusiasm, inspiration, pride, and challenge. Finally, absorption is characterized by being fully concentrated on and happily engrossed in one’s work, a sense that time passes quickly, and possibly finding it difficult to detach oneself from one’s work. Thus, work engagement comprises both well-being and motivation at work and thereby is a strong indicator of vitality at work. It has also been negatively related to burnout (exhaustion) over time [23], which in turn indicates a lack of vitality and mental energy. An extension of the original JD-R model further posits that job demands via burnout will impact health and work ability (energetic or health impairment process), whereas job resources via work engagement will enhance motivational outcomes such as job performance (motivational process) and reduce burnout [11]. 

In the work life context, sustainability is often referred to as an organizational-level concept, particularly a company’s inclusion of social and environmental concerns in business operations and in their interactions with stakeholders [24]. Thus far, the human dimension of sustainability and its work-related antecedents has gained much less research attention [25,26]. Sustainability from an employee perspective essentially refers to vitality and well-being, which have been regarded as the key elements of sustainable performance [19,27]. Many theorizations and models of sustainability at work also emphasize the importance of resources [19,28,29,30]. For example, the JD-R model captures important psychosocial work-related determinants of vitality and furthermore, of sustainable performance.

The JD-R model has received considerable support from many previous studies of a variety of occupations, claiming job demands to be the strongest predictors of burnout (or exhaustion), and job resources to be the main predictors of work engagement (or other motivational construct) [31]. Moreover, both cross-sectional and longitudinal studies have supported the revised and extended version of the model, further associating the job demands-burnout (exhaustion) and job resources-work engagement pathways with many important employee and organizational outcomes; for example, depressive symptoms and organizational commitment [32], duration and frequency of sickness absence [33,34], in-role and extra-role job performance [35], and health and work ability and organizational commitment [36]. Thus, empirical studies have supported the extended JD-R model, and we can expect job demands and job resources to be associated with elements of sustainable performance—being engaged and not exhausted, being healthy and productive—regardless of the employee’s contract type.

To the best of our knowledge, research on the role of employment contracts has rarely used the framework of the JD-R model. A study by van den Tooren and de Jong [37] found, for example, that contract type moderated the impact of job insecurity on both job satisfaction and general health, so that the negative relationships between job satisfaction and both outcomes were stronger among permanent than temporary workers. However, the main effects of job demands and resources on the outcomes separately in different contract groups have not been investigated.

### 1.3. The Present Study

By using linear regression analyses and dominance analysis (DA), this study aimed to compare the various determinants (job demands and job resources) of vitality at work (work engagement and exhaustion) in three employment contract groups: permanent, temporary and temporary agency workers. As job demands, we studied workload, emotional demands, physical demands, and job insecurity, which represent both the mental and physical demands of work. As job resources, we investigated resources at the task level—job control, job feedback; the interpersonal level—colleague support and supervisor support; and the organizational level—positive social climate. It is noteworthy that most of the studies in the existing literature on temporary versus permanent employment has focused on task-level resources such as autonomy, skill discretion, and participation in decision making. In the present study, we also studied interpersonal (colleague and supervisor support) and organizational (positive social climate) resources to gain a more comprehensive understanding of the potential differences between contract groups.

Following the predictions of the JD-R model, we formulated the hypothesis that, regardless of contract type, job resources are more strongly related to work engagement than job demands, and job demands in turn are more strongly related to exhaustion than job resources. In addition, we formulated two research questions. First, we investigated whether the three contract groups reported different levels of job resources, job demands, work engagement, and exhaustion. Second, our main goal was to study whether the relative importance of each job demand and job resource for work engagement and exhaustion was similar or different, depending on contract type. We did not formulate a hypothesis for these two research questions, as in this respect there are no a priori reasons to hypothesize on differences between these employment groups.

## 2. Materials and Methods

### 2.1. Sample and Procedure

In the present study, we used the data of the sixth European Working Conditions Survey (EWCS), collected in 2015 by Eurofound [38]. EWCS is conducted every five years using random samples of the workforce and focuses on occupation, working conditions and health. The target population for the EWCS consists of all residents in EU countries aged 15 or above and in employment at the time of the survey. A stratified (by region and degree of urbanization) multistage, random sample is drawn in each country, using individual-, household- and address-level registers. In each stratum, primary sampling units are randomly selected, in proportion to the size of the country. Subsequently, a random sample of households is drawn from each of these units. More details on sampling and overview of the general results in different countries can be found elsewhere [38].

The minimum sample size per country was 1000. The overall response rate was 43%, ranging from 11% in Sweden to 78% in Albania, and resulting in a total of 43,850 responses. All 28 EU Member States were included, as well as Norway and Switzerland. Five EU-associated countries were excluded on the basis of EWCS quality assurance [38]. The EWCS is representative of those aged 15 years and above (16 and above in Bulgaria, Norway, Spain and the UK) who are in employment and are resident in the country that is being surveyed. The final sample of the present study included those participants who were employed at the time of the survey and had a work contract of either limited or unlimited duration or who had a temporary employment agency contract (N = 28,042). Table 1 presents the demographic details of the participants in the three employment groups. 

### 2.2. Measures

The measurement used five-point Likert scales, ranging from 1 (strongly agree or always) to 5 (strongly disagree or never). We reversed all the scales so that the higher values referred to higher levels of work engagement, exhaustion, and each job resource and job demand.

Work engagement was assessed using three items from the Utrecht Work Engagement Scale (UWES) [22]: ‘At my work, I feel full of energy’ (vigor), ‘I am enthusiastic about my work’ (dedication), and ‘Time flies when I am working’ (absorption). Cronbach’s alpha was α = 0.71. Recently, a similar three-item version of the UWES was validated and shown to be psychometrically as sound as the nine-item version (UWES-3) [39,40]. 

Exhaustion was measured using one item from Maslach Burnout Inventory [41]: ‘I feel exhausted at the end of the working day’. This item correlated 0.80 with the MBI exhaustion subscale and 0.69 with the remaining four items of the subscale among 28,738 employees from an international burnout database [42]. Previous studies have used both the exhaustion scale [43,44] and the single item used in the present study as a proxy for burnout [42], but for the sake of clarity, in this paper, we use the term ‘exhaustion’.

Job control was measured using three items, for example: ‘You can influence decisions that are important for your work’. Cronbach’s alpha was 0.68.

Job feedback comprised two items, for example: ‘Your job gives you the feeling of work well done. The correlation between the two items was 0.54. 

Colleague support was measured using two items, for example: ‘Your colleagues help and support you’. The correlation between the two items was 0.71. 

Supervisor support was measured using six items, for example: ‘Your supervisor is helpful in getting the job done’. Cronbach’s alpha was α = 0.87. 

Positive social climate was assessed using five items, for example: ‘Employees are appreciated when they have done a good job’. Cronbach’s alpha was α = 0.83.

Workload was measured using two items, for example: ‘Does your job involve working at very high speed?’ The correlation between the two items was 0.64. 

Emotional demands comprised two items, for example: ‘Being in situations that are emotionally disturbing for you’. The correlation between the two items was 0.47.

Physical demands were measured using seven items, for example: ‘Are you exposed at work to high/low temperatures?’ Cronbach’s alpha was 0.82. 

Job insecurity was measured using one item: ‘I might lose my job in the next six months.’

In addition, in the regression models, our covariates were age, sex, highest level of education (ISCED), country including design weights to adjust for different selection probabilities, sectors of economic activity (NACE), and occupational groups (ISCO).

### 2.3. Analytical Strategy

We conducted two types of statistical analyses. First, we used linear regression analysis to separately investigate the associations between job demands and job resources, and work engagement and exhaustion in the three employment contract groups. The results of the linear regressions are presented as exponentiated regression coefficients (exp[β]), with 95% confidence intervals (CI). We adjusted all the linear models for age, sex, education, occupational group, sectors of economic activity and country. We weighted all the analyses with design weights to adjust for different selection probabilities of EWCS to control for the impact of country. The present dataset provided a unique possibility to control for the impact of country, as economic situation, country-specific rules and legislation may vary from country to country [1].

Second, we investigated the relative importance of different job demands and job resources for work engagement and exhaustion in the three groups by conducting dominance analysis (DA) to determine the most important contributors to work engagement and exhaustion [45]. We also reported the dominance value (domin), which means the proportion of each factor’s explained variance (%) of the variance explained by the whole model (100%). DA compares all independent variables in the model to each other and ranks them by their relative importance for predicting outcomes [46].

DA has been an underutilized multiple regression approach in work and organizational psychology, and its wider use has been advocated to gain an appropriate understanding of the role played by each predictor in a regression equation [45,46,47]. The advantage of DA is that it appropriately partitions variance to the various correlated predictors [47,48]. It also overcomes methodological difficulties such as the multicollinearity related to traditional regression models with several correlated predictors [45]. We used Stata 14.0 software (Stata Corporation, LLC College Station, TX, USA) and its DOMIN module [49] to conduct the analyses. 

Finally, as we constructed the job demand and job resource scales from the existing data, we tested the overall measurement model (confirmatory factor analysis) which specifies the pattern by which each measure is loaded on a factor [50]. The measurement model showed an acceptable model fit (χ^2^ (370) = 25711.98, CFI = 0.93, NFI = 0.93, SRMR = 0.031 and RMSEA = 0.044) and we detected no cross-loadings between different factors. These analyses indicated that the work characteristics could be distinguished from each other.

## 3. Results

### 3.1. Descriptive Results

The mean levels of work engagement and exhaustion in three different employment contract groups did not appear to differ considerably (Table 2). As regards the levels of job resources, the differences were also minor, except for job control, which was highest among the permanent workers (M = 3.3), the next highest among the temporary workers (M = 3.0) and the lowest among the temporary agency employees (M = 2.7). Temporary agency employees reported slightly higher levels of job demands, except for emotional demands (M = 2.0 vs. M = 2.5 in other two groups), than the other groups. The biggest difference concerned job insecurity, which was highest among the temporary agency workers (M = 3.3), followed by the temporary workers (M = 3.0) and clearly lowest among the permanent workers (M = 1.9). Generally, the temporary agency employees appeared to report less job resources and more job demands than the other two groups.

### 3.2. Results of Regression Analyses

After adjustment for various demographic and occupational factors as well as country, all job resources—job control, job feedback, colleague support, supervisor support, and positive social climate—were positively related to work engagement in all the employment contract groups (Table 3). In contrast, the associations between work engagement and job demands—workload, emotional demands, physical demands and job insecurity—were weakly negative or insignificant. 

The strengths of the associations between job characteristics and vitality at work in the different contract groups were also quite similar. For example, work engagement was positively associated with job feedback among the permanent employees [exp(coef) = 1.55, 95%CI 1.52–1.58], the temporary employees [exp(coef) = 1.53, 95%CI 1.46–1.60], and the temporary agency employees [exp(coef) = 1.69, 95%CI 1.50–1.90]. However, the negative association between work engagement and job insecurity was significant among the permanent employees [exp(coef) = 0.91, 95%CI 0.89–0.92] and the temporary employees [exp(coef) = 0.95, 95%CI 0.93–0.98] but insignificant among the temporary agency employees [exp(coef) = 1.01, 95%CI 0.92–1.11].

As regards exhaustion, we found the opposite pattern, as three job demands—namely workload, emotional demands and physical demands—were positively related to exhaustion in all three employment contract groups (Table 4). Job insecurity was only associated with exhaustion among the permanent workers [exp(coef) = 1.10, 95%CI 1.08–1.13] and was insignificant in the other contract groups. This finding is intriguing, as permanent employees reported the lowest levels of job insecurity. In addition, all five job resources were negatively associated with exhaustion among the permanent and temporary employees. Among the temporary agency employees, only job control was negatively associated with exhaustion [exp(coef) = 0.84, 95%CI 0.73–0.97], whereas the other job resources were insignificantly related to exhaustion. 

### 3.3. Results of Dominance Analysis

Table 5 reports the results of DA for work engagement. DA models explained 31.1% of the variance of work engagement among the permanent employees, 36.6% among the temporary employees and 38.7% among the temporary agency employees. All five job resources contributed more than job demands to work engagement. Of all the variance of work engagement explained by DA, job resources explained 94–95% in all three employment contract groups. 

Job feedback made the strongest contribution to work engagement in all three employment groups (37–41% of the explained variance) followed by positive social climate (15–23%) and supervisor support (13–22%). After these three job resources, colleague support (9–11%) and job control (7–13%) contributed most to work engagement. We found no major differences between the contract groups in the order of relative importance of the various job resources. Supervisor support contributed somewhat more to work engagement among the temporary agency employees (22%) than among the other two groups (13–14%).

Four job demands, age, sex, and country played a very minor or no role at all in explaining the variance of work engagement. Job insecurity explained 4% of the total variance explained by DA of work engagement among the permanent workers, whereas otherwise, job demands contributed to work engagement by only 0–2%, regardless of contract type. 

Table 6 displays the results of DA for exhaustion. The DA models explained 14.8% of the variance of exhaustion among both the permanent and temporary employees and 22.3% among the temporary agency employees. Generally, job demands accounted for 69–79% and job resources for 18–28% of the total variance in exhaustion. Workload made the strongest contribution to exhaustion in all three groups, accounting for 34–37% of the total variance explained by DA in exhaustion, followed by physical demands (12–26%) and emotional demands (7–19%). The fourth job demand, job insecurity, contributed from 4% (permanent workers) to 0% (temporary agency employees) to the total variance explained.

The results between different employment groups were more inconsistent for exhaustion than for work engagement. Physical demands were relatively less important for the permanent workers (12%) than for the temporary workers (23%) or those with temporary employment agency contracts (26%). In addition, for the temporary agency employees, emotional demands played a weaker role (7%) in exhaustion than for the other two groups (19% for the permanent workers and 17% for the temporary workers). Job control (negatively) contributed to exhaustion particularly among the temporary agency employees (12% vs. 6% for the permanent workers and 5% for the temporary workers). In addition, positive social climate (also negatively) contributed clearly less to exhaustion in this group (2%) than in the other groups (12% for the permanent workers and 9% for the temporary workers). Finally, sex (6%) and country (6%) contributed to exhaustion among the temporary agency employees, but not to work engagement and not in the other contract groups.

## 4. Discussion

The overall aim of the present study using the JD-R model and data on over 28,000 employees from 30 European countries was to investigate whether there were differences between the determinants of vitality at work of permanent, temporary and temporary agency employees. We found some differences in the mean levels of working conditions and the outcomes between the employment groups. The temporary agency workers in particular reported higher job demands and lower job resources, especially job control, than the permanent and temporary employees. In addition, the greatest difference concerned job insecurity, which was highest among the temporary agency workers and lowest among those with permanent jobs. The results of many previous studies on the differences between the mean levels of work characteristics and well-being have been very mixed, sometimes suggesting better working conditions and well-being among those with more stable jobs and sometimes the opposite or no differences [1,3,4,5,6,7,9,15,16,17]. 

However, instead of considering contract type as a ‘predictor’ of working conditions and vitality, our main goal was to compare the relationships between the determinants (job demands, job resources) of vitality at work (work engagement, lack of exhaustion) in the different contract groups. We found that these relationships were quite consistent across the groups. In addition, closer inspection of the relative importance of the different determinants of work engagement and exhaustion revealed a mainly similar pattern in the three contract groups, as we found that several job demands and job resources quite similarly contributed to work engagement and exhaustion, regardless of contract type. These findings were more robust, as we controlled for the impact of many background factors, such as country and various demographic factors. Below we discuss the results and theoretical implications in more detail.

### 4.1. Theoretical Implications

In this study, we aimed to bring together the theoretical framework of the JD-R model, a comparison of different employment contract groups, and the emerging field of human sustainability with its focus on vitality at work. The JD-R model is currently the most popular job design and occupational health psychological framework [31], yet it has only sparsely been used to compare different employment contract groups. However, human sustainability and sustainable performance have remained somewhat weakly defined concepts [51], and research on the impacts of different contract types has suffered from a lack of theoretical perspectives [4]. 

We operationalized vitality at work as work engagement and (lack of) exhaustion and studied their determinants—both job demands and job resources. The job demand-exhaustion (energetic process) and the job resource-work engagement (motivational process) pathways are known to be associated with better physical and mental health, work ability and productivity [32,33,34,35,36]. Therefore, although we did not investigate the further consequences of these two pathways in this study, we consider the JD-R model suitable for investigating vitality at work that can enhance sustainable performance, i.e., well-being, health, and productivity in different employment groups.

We found support for our hypothesis concerning the main assumption of the JD-R model [10,11,21], as in all the contract groups, all five job resources (job control, job feedback, colleague support, supervisor support, and positive social climate) were positively related to work engagement, whereas the four job demands played only a minor role in work engagement. Moreover, three job demands (workload, emotional demands and physical demands) were positively related to exhaustion. All five job resources were negatively related to exhaustion among the permanent and temporary employees, with the exception that job control was the only job resource significantly and negatively associated with exhaustion among the temporary agency employees. Previous studies comparing different employee groups have similarly found the same job demands and resources to be equally associated with outcomes such as work engagement, burnout [52], task satisfaction, work-related fatigue and organizational commitment [53] in different sectors.

To dig deeper into the comparison of the importance of different determinants of vitality at work, our goal was to use DA to identify the most important job demands and resources contributing to work engagement and exhaustion in each contract group and to compare differences in the rank order of these predictors for vitality at work. We found that, generally, in all the three employment groups (permanent, temporary and temporary agency workers), job resources were more important for work engagement and job demands for exhaustion, thus supporting our findings based on linear regression modelling and the JD-R model [11]. Regardless of the employment group, the five job resources contributed more than 90% to the explained variance of work engagement, whereas job demands, country, age, and gender contributed very little. Moreover, job feedback, followed by supervisor support and positive social climate made the strongest contribution in all the contract groups. Job feedback is one of the core job characteristics in the job characteristics theory, which proposes that job feedback increases work motivation, sense of meaningfulness and job satisfaction [54]. Job feedback may be particularly important for work engagement, because it provides information on one’s activities as well as a sense of accomplishment and competence, which are all important ingredients for engagement at work. 

Interestingly, interpersonal and organizational job resources, such as supervisor support and positive social climate, also proved to be important contributors to work engagement in all the employment groups. Previous studies have often investigated the differences in task-level job resources (autonomy, participation in decision making, skill variety) in different employment groups [5]. The present study suggests that interpersonal and organizational job resources may also be important for work engagement among those with less stable contracts, even for temporary agency employees. Future research could investigate even more different job resources in non-standardized contract groups.

As regards the other dimension of vitality at work—exhaustion—we found that workload clearly contributed the most to exhaustion in all the employment groups. This was followed by physical demands (particularly in the temporary and temporary agency jobs) and emotional demands (particularly in the permanent and temporary jobs but less in the temporary agency jobs). These three job demands accounted more for exhaustion than the other variables, except for job control, which was more relevant (protective factor) to exhaustion than emotional demands among the temporary agency workers. Workload and having too much to do in a given time has been consistently and strongly related to burnout—particularly to its core dimension, exhaustion [55]. In addition to job demands, positive social climate among the permanent and temporary workers, and job control among the temporary agency workers contributed to (less) exhaustion. These findings suggest that despite similarities, partly different resources/demands are important for different employment contract groups.

Interestingly, although the mean level of job insecurity was lowest among permanent workers, the relative importance of job insecurity for work engagement and exhaustion was highest in the permanent contract group. This suggests that permanent employees may have more at stake in their work and therefore job insecurity may especially threaten vitality in this group. This finding aligns with some previous studies which have also shown that permanent employees may suffer more from job insecurity than temporary workers [56,57,58]. The explanation for this may lie in the psychological contract theory, which differentiates between relational psychological contract and transactional contract [58]. The first one comprises a deeper socio-emotional exchange relationship, i.e., job security in exchange for loyalty and commitment, and this type of contract is more typical for permanent employees. The latter type of contract is characterized by economic and short-term exchange of benefits and contributions, such as getting paid for attendance, which is likely to be more typical for temporary employees. As job security is the key element of a relational psychological contract, its violation is likely to be more harmful for permanent than for temporary employees, although temporary employees usually experience more job insecurity. Indeed, De Cuyper and De Witte [58] found that job insecurity had more negative consequences (in terms of job satisfaction and organizational commitment) for permanent than for temporary employees, because they engaged more in relational psychological contracting. 

Similarly, although the mean level of job control was lowest among the temporary agency workers, job control was particularly important for (lack of) exhaustion among the temporary agency employees. At the same time, other job resources (colleague and supervisor support and positive social climate) contributed more to the work engagement of the temporary agency employees than job control. Thus, not only may partly different resources/demands be important for different employment groups, but their relevance for a particular employment group may be different depending on the outcome. In addition, it is notable that the temporary agency employees reported lower levels of the job resources that were most important for their vitality at work, thus making them a vulnerable employment group. This finding deserves more attention in future studies, hopefully using longitudinal designs.

### 4.2. Limitations

A major strength of this study was that we could use a large, representative sample consisting of 28,042 employees from 30 European countries. Except for one study in seven countries comparing employment contracts from a psychological contract perspective by Guest, Isaksson, & De Witte [17], most previous studies on the quality of working conditions and employee well-being have been conducted in one country. Due to our large, multi-country dataset, we were also able to control for the impact of many confounding factors, particularly the impact of country, as many societal and legal issues concerning employment contracts vary between countries. Another strength was that in addition to linear regression analyses, we employed a rather rarely used statistical method—dominance analysis—which is particularly suitable for comparing the relative importance of different predictors for the outcomes of interest [45,46,47].

Besides its strengths, this study has also several limitations. First, it is based on self-reports. Although it is difficult to find objective measures for experiencing vitality: engaged and not fatigued at work, it would have been interesting to include objectively measured or other-rated working conditions [10]. Another interesting future option would be to compare the relative importance of pay level and pay level satisfaction as job resources for work engagement and exhaustion in different contract groups. The study’s cross-sectional design is its second major limitation, as it prevented the study of the long-term impacts of job demands and job resources in the different contract groups. Although abundant research on different contract types exists, there is a clear need for longitudinal comparisons of different contract groups. Another limitation related to the cross-sectional design in the present study is that it was not possible to test reversed or reciprocal associations between the study variables. Several studies have found reciprocal impacts between, for instance, job resources and work engagement [59,60], and job demands and exhaustion [61]. A recent meta-analysis of the JD-R model also confirmed these findings by showing that work engagement and burnout are not only influenced by various job demands and resources: they may also impact them [31]. An interesting future avenue of research in this field would be to study, for instance, how potential positive reciprocal job resource-work engagement cycles could build better career paths among different contract groups.

A third limitation of this study is that we could only measure five different job resources and four job demands. There are more that we could have studied [62]. For instance, skill utilization has been found to be a particularly important determinant for many employee outcomes [53]. Fourth, today’s work life has more non-standardized employment contracts than those included in our study. In the future, more attention should be paid to the specific job demands and job resources—and also to the lack of demands and resources—and their relationships with vitality at work of those working in platforms, for instance [63]. Finally, although our study examined 30 countries, they were all European. Thus, we do not know the extent to which our results can be generalized to other parts of the world.

### 4.3. Practical Implications

Different types of non-traditional and non-standardized employment contracts have become more common in recent decades. For sustainable performance in organizations across different industries, it is of utmost importance that employees, regardless of their contract types, have enough resources available and that their job demands are not too high, so that they can experience vitality at work. Several previous studies suggest that engaged employees perform better, are mentally and physically healthier, and also manage to better balance work and family [32,33,34,35,36]. According to this study, feedback, i.e., being able to see the results of one’s contributions and experience a feeling of work well done, was the most important driver of work engagement in all employment groups. Feedback is an experience that can be boosted in at least two ways. One is by means of proactively crafting one’s job by, for example, paying attention to its purpose and to one’s accomplishments while working and in this way increasing meaningfulness and engagement at work [64]. The other way, in addition to this bottom–up approach, is a traditional top–down practice, for instance, regularly giving prompt, specific and improvement-oriented positive feedback to employees, and not forgetting those whose contract may only have a limited duration. When job feedback is increased, work engagement grows. 

In addition to job feedback, this study found many other resources that can be increased to boost work engagement and, to some extent, also to decrease exhaustion, regardless of contract type. Our results also suggest that temporary agency workers partly lack the job resources that would be most beneficial for their vitality. Therefore, they may benefit both from supervisor support (to increase work engagement) and job control (to decrease exhaustion) even more than other employment groups. The results of DA also revealed that physical demands contributed particularly to the exhaustion of temporary and temporary agency employees, and therefore in addition to reducing workload efforts to improve the physical work environment may not only prevent from physical health problems and injuries, but also sustain vitality. Organizations may also benefit from understanding that for permanent employees, job security is even more important, at least in terms of vitality, than for other contract groups. Therefore, it can be valuable to focus on relational psychological contracting and avoid insecurity, raising issues as much as possible.

## 5. Conclusions

In conclusion, this study used a large-scale European dataset from 30 countries to investigate the relationships between four job demands and five job resources and vitality at work—conceptualized as feeling engaged and not exhausted at work—among permanent, temporary and temporary agency workers. Overall, after controlling for many confounders, job resources were the main antecedents of work engagement, and job demands the antecedents of exhaustion in all the employment groups. In addition, job feedback made the strongest contribution to work engagement, and workload the strongest contribution to exhaustion across the employment groups, suggesting that to boost sustainable performance in organizations, both job demands and job resources must be impacted. Notably, temporary agency employees reported less job resources and more job demands and would benefit more than other groups from supervisor support and job control to stay vital at work. All in all, however, despite some differences in the determinants of vitality at work, these groups are likely to benefit from similar job resources and job demands that are not too high, particularly quantitative workload.

## Figures and Tables

**Table 1:** Characteristics of the study population.

Variables/Contract Groups	Permanent Employees (n = 24,282)		Temporary Employees (n = 3380)		Temporary Agency Employees (n = 380)
Gender					
Men	11,468		1541		198
Women	12,789		1838		182
Age groups					
< 30 years	3056		1034		122
30–40 years	5834		947		91
40–50 years	7023		695		81
50–60 years	6570		494		60
> 60 years	1645		172		22
Education					
Early childhood education	68		25		3
Primary education	479		129		19
Lower secondary education	2661		466		84
Upper secondary education	10,211		1379		172
Post-secondary non-tertiary education	2017		338		15
Short-cycle tertiary education	2595		263		23
Bachelor or equivalent	3189		410		43
Master or equivalent	2743		319		17
Doctorate or equivalent	233		44		2

**Table 2:** Means and standard deviations (SD) across employment contract groups.

Variables/Contract Groups	Permanent Employees (n = 24,282)		Temporary Employees (n = 3380)		Temporary Agency Employees (n = 380)	
	Mean	SD	Mean	SD	Mean	SD
Employee well-being						
Work engagement	3.9	0.7	3.8	0.8	3.8	0.9
Exhaustion	3.1	1.0	3.2	1.1	3.2	1.2
Job resources						
Job control	3.3	1.0	3.0	1.1	2.7	1.1
Job feedback	4.3	0.8	4.2	0.9	4.1	0.9
Colleague support	4.3	0.7	4.2	0.8	4.1	0.9
Supervisor support	3.9	0.9	3.9	0.9	3.8	1.00
Positive social climate	3.9	0.8	3.9	0.8	3.8	0.9
Job demands						
Workload	3.6	1.8	3.7	1.9	3.9	2.0
Emotional demands	2.5	1.4	2.5	1.5	2.0	1.4
Physical demands	1.8	0.9	1.8	1.0	2.0	1.0
Job insecurity	1.9	1.2	3.0	1.5	3.3	1.4

**Table 3:** Associations between job resources and job demands (separately in the models) with work engagement among various types of labor contract. The models are adjusted for age, sex, country including design weights to adjust for different selection probabilities, sectors of economic activity (NACE), and occupational groups (ISCO).

	Work Engagement					
	Permanent Employees (n = 24,282)		Temporary Employees (n = 3380)		Temporary Agency Employees (n = 380)	
	Regression Coefficient	95%CI	Regression Coefficient	95%CI	Regression Coefficient	95%CI
Job resources						
Job control	1.25	1.23, 1.27	1.25	1.21, 1.30	1.17	1.03, 1.33
Job feedback	1.55	1.52, 1.58	1.53	1.46, 1.60	1.69	1.50, 1.90
Colleague support	1.33	1.30, 1.36	1.37	1.30, 1.45	1.28	1.09, 1.51
Supervisor support	1.34	1.31, 1.36	1.41	1.34, 1.47	1.49	1.32, 1.68
Positive social climate	1.39	1.36, 1.42	1.53	1.46, 1.60	1.56	1.36, 1.78
Job demands						
Workload	0.98	0.97, 0.99	0.94	0.92, 0.97	0.97	0.91, 1.03
Emotional demands	0.97	0.95, 0.98	0.96	0.93, 0.99	0.94	0.85, 1.03
Physical demands	0.98	0.97, 1.00	0.93	0.89, 0.97	1.03	0.88, 1.21
Job insecurity	0.91	0.89, 0.92	0.95	0.93, 0.98	1.01	0.92, 1.11

**Table 4:** Associations between job resources and job demands (separately in the models) with exhaustion among various types of labor contract. The models are adjusted for age, sex, country including design weights to adjust for different selection probabilities, sectors of economic activity (NACE), and occupational groups (ISCO).

	**Exhaustion**					
	Permanent Employees (n = 24,282)		Temporary Employees (n = 3380)		Temporary Agency Employees (n = 380)	
	Regression Coefficient	95%CI	Regression Coefficient	95%CI	Regression Coefficient	95%CI
Job resources						
Job control	0.90	0.87, 0.92	0.87	0.82, 0.92	0.84	0.73, 0.97
Job feedback	0.86	0.83, 0.88	0.90	0.84, 0.96	1.08	0.87, 1.34
Colleague support	0.89	0.86, 0.92	0.91	0.84, 0.98	0.99	0.83, 1.19
Supervisor support	0.82	0.80, 0.85	0.85	0.80, 0.91	0.94	0.78, 1.13
Positive social climate	0.76	0.74, 0.78	0.75	0.70, 0.81	0.99	0.82, 1.21
Job demands						
Workload	1.18	1.16, 1.19	1.22	1.18, 1.25	1.20	1.11, 1.30
Emotional demands	1.16	1.14, 1.18	1.14	1.10, 1.20	1.20	1.07, 1.33
Physical demands	1.27	1.24, 1.30	1.40	1.32, 1.48	1.34	1.11, 1.63
Job insecurity	1.10	1.08, 1.12	1.02	0.97, 1.07	1.01	0.89, 1.15

**Table 5:** The position in the ranking of dominance analysis (Domin rank) and standardized dominance estimates (Domin = % R^2^ explained) for work engagement.

Work Engagement						
	Permanent Employees (n = 24,282)		Temporary Employees (n = 3380)		Temporary Agency Employees (n = 380)	
	Domin Rank	Domin	Domin Rank	Domin	Domin Rank	Domin
Sex	9	0%	10	0%	12	0%
Age	12	0%	11	0%	6	2%
Country	11	0%	12	0%	8	1%
Job resources						
Job control	4	13%	5	7%	5	8%
Job feedback	1	37%	1	41%	1	38%
Colleague support	5	11%	4	9%	4	10%
Supervisor support	3	14%	3	13%	2	22%
Positive social climate	2	19%	2	23%	3	15%
Job demands						
Workload	8	0%	7	2%	9	1%
Emotional demands	10	0%	9	0%	11	0%
Physical demands	7	1%	6	2%	10	1%
Job insecurity	6	4%	8	1%	7	1%
		100%		100%		100%

**Table 6:** The position in the ranking of dominance analysis (Domin rank) and standardized dominance estimates. (Domin = % R^2^ explained) for exhaustion.

Exhaustion						
	Permanent Employees (n = 24,282)		Temporary Employees (n = 3380)		Temporary Agency Employees (n = 380)	
	Domin Rank	Domin	Domin Rank	Domin	Domin Rank	Domin
Sex	8	3%	7	2%	6	6%
Age	11	0%	11	0%	11	0%
Country	12	0%	12	0%	5	6%
Job resources						
Job control	5	6%	5	5%	3	12%
Job feedback	9	2%	9	1%	10	1%
Colleague support	10	2%	10	1%	7	2%
Supervisor support	6	6%	6	3%	9	1%
Positive social climate	4	12%	4	9%	8	2%
Job demands						
Workload	1	34%	1	37%	1	36%
Emotional demands	2	19%	3	17%	4	7%
Physical demands	3	12%	2	23%	2	26%
Job insecurity	7	4%	8	2%	12	0%
		100%		100%		100%
